# Tuning Structure and Dynamics of Blue Copper Azurin Junctions via Single Amino-Acid Mutations

**DOI:** 10.3390/biom9100611

**Published:** 2019-10-15

**Authors:** Maria Ortega, J. G. Vilhena, Linda A. Zotti, Ismael Díez-Pérez, Juan Carlos Cuevas, Rubén Pérez

**Affiliations:** 1 Departamento de Física Teórica de la Materia Condensada, Universidad Autónoma de Madrid, E-28049 Madrid, Spain; maria.ortega@uam.es (M.O.); linda.zotti@uam.es (L.A.Z.); juancarlos.cuevas@uam.es (J.C.C.); 2 Department of Physics, University of Basel, Klingelbergstrasse 82, CH-4056 Basel, Switzerland; 3 Condensed Matter Physics Center (IFIMAC), Universidad Autónoma de Madrid, E-28049 Madrid, Spain; 4 Department of Chemistry, Faculty of Natural & Mathematical Sciences, King’s College London, Britannia House, 7 Trinity Street, London SE1 1DB, UK; ismael.diez_perez@kcl.ac.uk

**Keywords:** biomolecular electronics, Azurin, single molecule, solid-state junction, molecular dynamics, protein adsorption, electronic transport, single-point-mutation

## Abstract

In the growing field of biomolecular electronics, blue-copper Azurin stands out as one of the most widely studied protein in single-molecule contacts. Interestingly, despite the paramount importance of the structure/dynamics of molecular contacts in their transport properties, these factors remain largely unexplored from the theoretical point of view in the context of single Azurin junctions. Here we address this issue using all-atom Molecular Dynamics (MD) of Pseudomonas Aeruginosa Azurin adsorbed to a Au(111) substrate. In particular, we focus on the structure and dynamics of the free/adsorbed protein and how these properties are altered upon single-point mutations. The results revealed that wild-type Azurin adsorbs on Au(111) along two well defined configurations: one tethered via cysteine groups and the other via the hydrophobic pocket surrounding the Cu${}^{2+}$. Surprisingly, our simulations revealed that single amino-acid mutations gave rise to a quenching of protein vibrations ultimately resulting in its overall stiffening. Given the role of amino-acid vibrations and reorientation in the dehydration process at the protein-water-substrate interface, we suggest that this might have an effect on the adsorption process of the mutant, giving rise to new adsorption configurations.

## 1. Introduction

Blue-copper proteins, such as Azurin, represent the test-bench system in the field of biomolecular electronics [1,2,3,4,5,6]. Their electron-transport properties were analyzed via experiments based on self-assembled monolayers [5], as well as single-protein wires using Scanning Tunneling Microscopy (STM) [2,3]. Strikingly, the measured conductance values were found to be comparable with that of much shorter alkanedithiol chains [3] (up to three times). Moreover, the possibility of tuning their electron-transport mechanism by inserting mutations [2] or modifying the metal-protein coupling [5] was revealed. This prompted several theoretical studies aimed at understanding the electronic properties of these systems [7,8]. However, not much is known about the dynamics and the structure of these proteins when adsorbed on a metal surface [2,9]. This information is crucial to rationalize the preceding findings, since the conditions used, for instance, during experimental measurements such as STM (in either ambient condition or under electrochemical control) often hinder atomically resolved imaging of the molecule [10]. In fact, some of the experimental strategies developed to detect the nature of junctions between metals and small organic molecules (such as the analysis of conductance plateaus upon pulling of the junction) cannot be directly applied to proteins, as they could unfold and lose their properties during processing [2]. Consequently, important Azurin structural and dynamical parameters such as its interaction with the electrodes, or its orientation relative to them, cannot be straightforwardly gathered from the experiments [9].

Molecular Dynamics (MD) simulations of the protein dynamics may allow us to gain further insight into the structure and behavior of the metal-protein-metal junctions [11,12,13,14]. These studies can provide information regarding protein orientation, and whether the protein folding is preserved or affected by the interaction with the electrodes [14]. This method was already used in the past to study the structure and dynamics of the Azurin protein free solved in water [15,16] as well as its anchoring to a gold surface via its cysteines [17,18,19]. However, to our knowledge, the systematic analysis of the free adsorption process of this protein onto gold surfaces, i.e., with no preferential anchorage point assumed a priori, has remained elusive. Such free-adsorption study may allow us to unravel not only the characteristics of the Azurin-gold electrode interaction beyond cysteine-gold tethering (e.g., adsorption configurations, protein dynamics, etc.), but also other factors which may influence the protein-surface interaction.

The role of structural modifications as well as the stability of protein-metal contacts may also be explored through the introduction of single amino-acid mutations in the protein chain [2]. Such mutations have been widely used to increase the stability of the junction by inserting residues (for instance cysteine) which are known to strongly bind to gold [2]. Alternatively, such mutations may also alter the adsorption configurations, e.g., by favouring certain adsorption orientations with respect to others. Additionally, as mentioned above, such mutations can also change the electronic structure properties of the protein eventually altering the transport process mechanism [2]. These observations motivate the need for a better understanding of the role of these mutations in the structural properties of an Azurin-metal junction [2]. Moreover, the protein dynamics and how it is altered upon a mutation becomes an essential ingredient to understand the details of the protein/electrode adsorption. Interestingly, several reports [20,21] show that point-like mutations which retain the protein’s original structure strongly affect its flexibility/vibrations, in some cases almost doubling its stiffness [21,22]. These observations are general as they hold not only for the Azurin [20,23,24,25], but also in many other proteins [26,27,28,29,30,31]. Thus, tuning protein dynamics via point-like mutations may offer yet another way of controlling their adsorption dynamics and configurations. This is particularly appealing in biomolecular electronics as any significant change in its dynamics will certainly affect its coupling to the electrodes [18], which in turn could modify the electron transport mechanism through the metal-Azurin-metal junction [5]. Therefore, it stands clear that a detailed comparison between the structure and dynamics of wild-type Azurin and selected mutants during adsorption on a gold substrate will allow us to unravel the atomistic processes involved in the tuning of its electronic transport via mutations [2,7].

In this work, we perform long MD-simulations (reaching up to 0.5 μs for some of them) to analyze the structure and dynamics of a wild type Azurin from the *pseudomonas aeruginosa* in solution as well as its adsorption to Au(111). Furthermore, we explore how the structure and dynamics are affected via the introduction of single amino-acid mutations. To that aim, we first simulate the free dynamics in solution of three different mutated Azurin structures and compare it with the wild-type form. These three mutated structures are based on substituting a single amino-acid of the protein chain (K41, L120, and S89) by a cysteine, i.e., the K41C, L120C and S89C mutations. A detailed analysis of the fluctuations per residue in these three mutated structures reveals that the introduction of mutations quenches the flexibility of some turn regions of the protein, leading to an overall stiffening of the Azurin structure. We then test if this reduction of the flexibility affects the protein adsorption process by comparing the adsorption dynamics on a gold substrate of the wild-type and K41C proteins. For both Azurin variants, we simulate the adsorption on a Au(111) surface starting from four different protein orientations each, to allow a wider exploration of the possible adsorption configurations. The obtained results show that the wild-type Azurin structure adsorbs on the gold substrate preferentially in two different configurations: lying-down with the cysteines in contact with the surface or anchoring via the hydrophopic patch. This is possible thanks to the enhanced mobility shown by this protein allowing it to reorient its structure during adsorption. In contrast, the K41C mutant presents a smaller capability for self-reorienting during adsorption, resulting in different final adsorption configurations for each of the four initial protein orientations. The analysis of the fluctuations per residue in the free proteins reveals a stiffening effect induced by the presence of the mutations. Based on the important role of the amino acid vibrations and reorientation in the dehydration process at the protein-water-substrate interface during the adsorption process [32], we suggest a link between the stiffening and the different adsorption behavior of the mutants compared to wild type Azurin.

## 2. Methods

### 2.1. Atomic-Level Models and Force Fields

**Figure 1:**
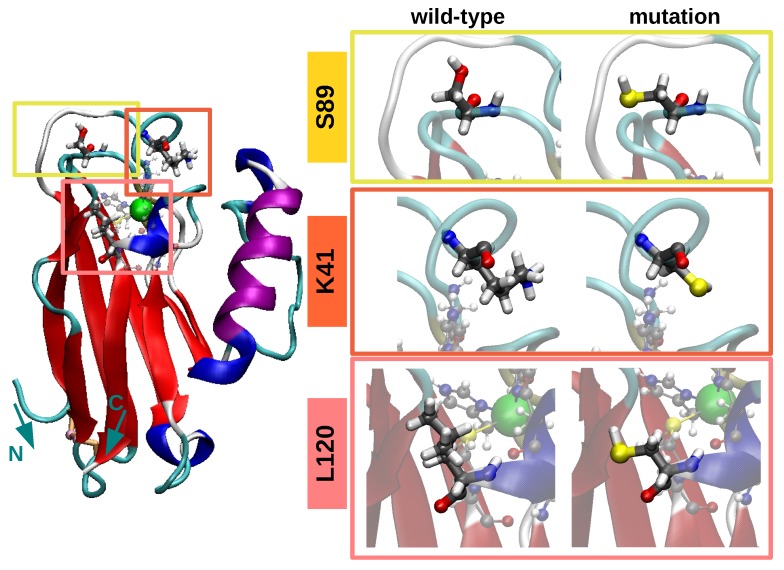
**Initial configuration of the Azurin proteins.** The Azurin is represented with its secondary structure: β-sheet (red), α-helix (purple), 310-helix (dark-blue), turns (cyan), and random-coils (white). The copper(II) ion is shown using its van-der-Waals representation in an opaque green color, and its coordination residues are represented with a ball-stick model. The disulfide bridge and the main chain of the two cysteines which formed it are colored in light orange. The sulfur atoms of these two cysteines are highlight in pink. The position of the three mutated residues (lysine 41, leucine 120 and serine 89) and the initial configuration (prior to minimization stages) of their side-chains in the wild-type and mutated proteins are here also indicated (orange, pink and yellow respectively). The distance between the Cu ion and the alpha carbon of the K41/L120/S89 mutated amino-acid is $d_{Mut-Cu}∼9.1$ Å/8.9 Å/11 Å.

In this work we considered five different proteins: wild-type Azurin, Apo Azurin and three mutants. The X-ray crystallographic structure of Azurin was obtained from the protein data bank [33] with the PDB code 4AZU [34]. Protons were added to the protein structure according to the calculated ionization states [35] of its titratable groups at a pH of 4.5, in accordance with recent experiments [2]. The Apo initial structure consisted of simply removing the copper ion from the crystallographic structure of Azurin. The three Azurin mutants here considered were prepared by replacing a given amino-acid (lysine 41, leucine 120 and serine 89) by a cysteine. This particular mutation is expected to promote the anchoring of the newly added cysteine to the gold contacts [2]. The residue replacement was performed changing the amino-acid type and removing the side-chain of the mutated amino-acid (lysine 41, leucine 120, serine 89) in the wild-type protein PDB with a text editor. The position of the atoms of the new side-chain was selected in agreement with the CYS ligand structure extracted from the protein data bank [33] (see the side-chain conformation of the mutated residues in Figure 1). Please note that although all mutations are in the vicinity of the copper(II) ion, they are located at different distances from it, see Figure 1 and Figure S1. In the L120C and K41C, the mutation is located in the second coordination sphere of the Cu atom ($d_{Mut-Cu}\;∼\;9$ Å) while in the S89C the mutation is in a flexible coil near the Azurin β-barrel at a distance of $d_{Mut-Cu}\;∼\;11$ Å from the copper(II) ion (see Figure 1). These relative positions between the mutated amino-acid and the Cu ion are maintained during the simulations in both the wild-type and mutated proteins as shown in Figure S2. The net charge of the resulting structures is zero for the wild-type, L120C and S89C, and −1 for the K41C and Apo. In that last two cases a Na${}^{+}$ counter-ion was added to neutralize the net charge of the system.

The surface used to study the protein adsorption is a Au(111) three atomic layers-thick slab. The initial cell used for creating this surface was a hexagonal cell with the lattice parameter of the Au(111) (2.9Å). Once the three-layer slab was created, it was truncated to get a rectangular slab with dimensions $8\times 8\;{\mathrm{nm}}^{2}$ in the x-y directions. A minimization of the surface structure alone was performed before the adsorption simulations to prevent steric clashes. The Au(111) lattice parameter value was not affected during this preliminary simulation. The positions of the atoms in the lowest layer were fixed during the MD runs using a harmonic restraint of 5 kcalmol${}^{-1}$.

The ff14SB force field [36] was used to describe all standard amino-acids present in the Azurin. The inter-atomic potentials of the copper(II) ion and its corresponding 5 ligands were described using a quantum mechanically derived force field [37]. This force-field includes both bonded and non-bonded terms between the Cu atom and its 5 ligands (see Figure S1) and was widely used to model the blue-copper Azurin protein [2,38,39,40,41]. In particular, recent experiments [39] showed how early stages of mechanical unfolding of this protein are well described by this force-field. The gold surface was described using the CHARMM-METAL [42,43] force field, which is thermodynamically consistent with the force field used to describe the protein and was successfully employed to study similar inorganic-bio-molecular interfaces [43,44]. All simulations were performed in water. These were explicitly modeled using the TIP3P force field [45]. The Joung/Cheatham parameters were used to describe the sodium counter-ions [46,47].

### 2.2. Molecular Dynamic (MD) Simulation Details

All the simulations were performed using the AMBER14 software suite [48] with NVIDIA GPU acceleration [49,50,51]. We used periodic boundary conditions (PBC) with a rectangular box larger enough to prevent the interaction between the protein and its periodic images (see Figure S3). Particle mesh ewald (PME), with a real–space cutoff of 10 Å, was used to account for long–range electrostatic interactions. Van-der-Waals (VdW) contacts were truncated at the real space cutoff of 10 Å. In all the simulations, the temperature of the system was adjusted by means of a Langevin thermostat [52] with a friction coefficient of γ=1 ps${}^{-1}\;$. For the simulations performed in the NPT ensemble (see Simulation Protocols), a Berendsen barostat [53] with a relaxation time of $t_{p}\;=\;1$ ps was used. The SHAKE [54] algorithm was used to constrain bonds containing hydrogen, thus allowing us to use an integration step of 2 fs. Coordinates were saved every 1000 steps.

### 2.3. Simulation Protocol for Azurin in Water

Unrestrained molecular dynamics simulations in water were performed for the five Azurin proteins here considered, i.e., wild-type, Apo, K41C, S89C and L120C. The simulation protocol consisted of four stages detailed below: (1) We prepared the system by embedding the protein in water in such a way that the minimum solute−water distance is 1 Å, thus resulting in a system with dimensions ∼72 Å×66 Å×70 Å. (2) We energy minimized the structures using a combination of steepest descent and conjugate gradient methods to avoid steric clashes; (3) We heated up the system from 0 to 300 K with a 2 ns NPT simulation to ensure a well-characterized water distribution at T=300 K and P=1 atm; (4) We performed the MD production runs in the NVT ensemble. We simulated the dynamics of the wild-type, Apo and K41C during 500 ns, and the dynamics of the L120C and S89C during 300 ns. We did not continue these last two simulations up to 500 ns (L120C, S89C) as we did not observe any major difference in the evolution of the structure (see Figure S4) and the fluctuations (see Figure S5) of the wild-type and K41C proteins from 300 to 500 ns of simulation. As shown in Figure S6, the evolution of the total energy of the system was stable during the last ∼200 ns for the five cases indicating that the simulations are sufficiently long to obtain thermally equilibrated structures.

### 2.4. Simulation Protocol for Azurin Adsorption

We simulated the adsorption process of two different proteins (wild-type and K41C) on a Au(111) slab. The simulation protocol was composed by the same four stages used in the unrestrained simulations aforementioned. The only two differences with respect to the previous protocol are detailed below. *Firstly*, the system preparation (step 1) was not the same. In the adsorption simulations, the Azurin is positioned above the Au(111) surface along four different initial orientations (see Figure S7a). The choice of these orientations is based on the tertiary structure of the protein. Namely, considering the Azurin as a cylinder whose main axis passes through the center of the β-sheet barrel then this axis can be oriented standing vertically over the surface (O2, O4 see Figure S7a) or parallel to the surface (O1, O3 see Figure S7a). Moreover, considering the strong interaction between cysteine groups and Au [55,56,57], we distinguished between the vertically standing Azurin with the cysteines close to the surface (O2, see Figure S7a) or further away from it (O4, see Figure S7a). The same distinction holds for the Azurins aligned parallel to the surface, i.e., O1/O3 (close/far) shown in Figure S7a. Regardless of the initial orientation, the initial protein-surface distance is ∼1 nm, see Figure S7a. This provides enough freedom for the protein to reorient itself if it must prior to its adsorption. The whole protein-surface complex was embedded in water, resulting in a system with dimensions ∼108 Å×108 Å×80 Å. *The second difference* concerns the duration of the production runs (step 4), as we simulated the adsorption dynamics for both wild-type and K41C proteins during 150 ns of NVT simulation. As shown in Figure S7b, the evolution of the total energy of the system is stable during the last ∼70 ns of simulation regardless the initial Azurin-gold configuration used, thus indicating that the simulations are sufficiently long to obtain thermally equilibrated adsorption configurations.

### 2.5. Trajectory Analysis

To characterize the protein structural stability we evaluated the following quantities: the root-mean-square deviation (RMSD) of the protein backbone [58], the protein radius of gyration($R_{g}$) [58], and the protein secondary structure content [59,60]. The reference structure used for computing the RMSD was the Azurin crystallographic structure. Moreover, to characterize the protein adsorption configurations we computed the evolution of the contact surface area (CSA) between the protein and the gold substrate. The latter is calculated using the following definition:

(1) $$CSA\left(t\right)=\frac{1}{2}\left(SA_{P}\left(t\right)+SA_{S}\left(t\right)-SA_{P-S}\left(t\right)\right)\;,$$

where the time dependent solvent-accessible-surface-area SA(t) [61] was calculated for the Azurin protein ($SA_{P}\left(t\right)$), the Au substrate ($SA_{S}\left(t\right)$), and the protein-substrate combined system ($SA_{P-S}\left(t\right)$). Additionally, to characterize the fluctuation of the protein during our MD simulations, we computed in all the cases the root-mean-square fluctuation (RMSF) of its residues [58]. That magnitude was computed considering only the protein dynamics that is in energetic equilibrium, i.e., the last 200 ns of the unrestrained Azurin dynamics in solution (see Figure S6) and the last 70 ns of the Azurin adsorption simulation (see Figure S7). The reference structure used for calculating the RMSF was the energetically equilibrated averaged Azurin configuration obtained in each simulation. The RMSF was calculated following a two-step procedure: (1) We aligned the Azurin trajectory in energetic equilibrium to its corresponding averaged configuration to evade translational and rotational effects in its RMSF value; (2) We compute the RMSF by comparing the aligned Azurin trajectory with its corresponding averaged configuration.

## 3. Results and Discussion

### 3.1. Equilibrium Structure of Unrestrained Wild-Type and Its Mutants in Water

**Figure 2:**
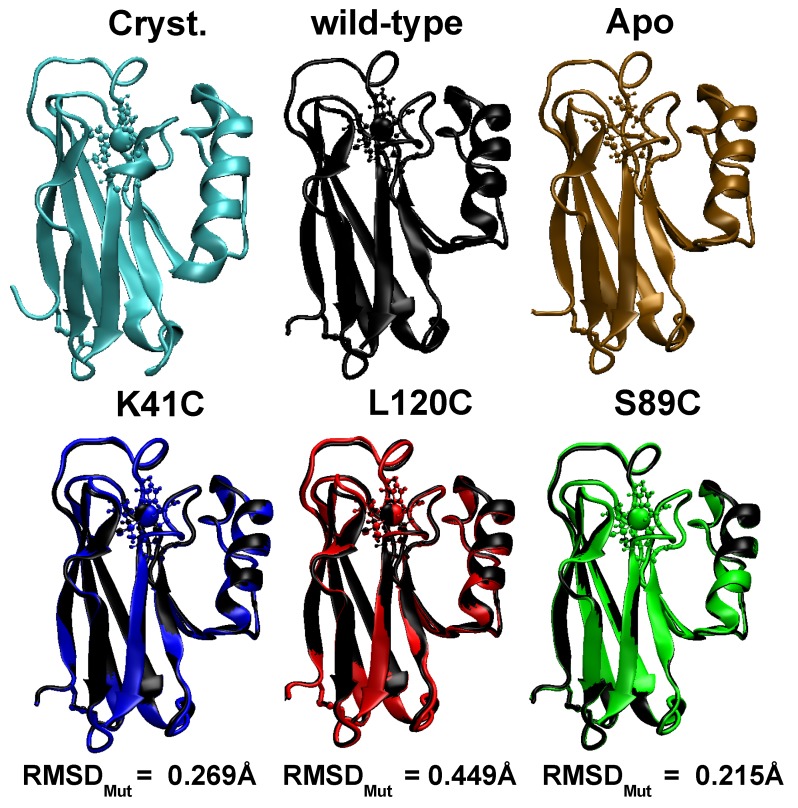
**Energetically equilibrated averaged configurations obtained for the unrestrained dynamics in water of the wild-type (black), the Apo (brown), the K41C (blue), the L120C (red) and the S89C (green) proteins.** The crystallographic structure [34] is also shown for comparison (cyan). The representation used is the same as in Figure 1. Please note that the three mutant configurations are aligned with the wild-type configuration (black) and superposed to it. The difference is quantified by the RMSD between both average configurations (shown as $RMSD_{Mut}$).

In Figure 2, we represent the time averaged configurations of the five Azurin structures (wild-type, Apo, three mutants) obtained in an unrestrained MD simulation in water. The wild-type (black) and Apo (brown) proteins are almost identical to the crystallographic one [34], in agreement with previous results [62]. A quantitative estimation of this similarity is provided through the time evolution of their RMSD shown in Figure S4. There, we find that when the system is in thermodynamic equilibrium (i.e., in the last 200 ns) the standard deviation of the RMSD is smaller than ∼0.2 Å. Interestingly, this shows that Azurin is a relatively stiff molecule in contrast to the most abundant plasma proteins such as IgG and BSA [32,63,64,65], which is certainly a desirable feature for its incorporation in biomolecular solid state devices. Furthermore, we also note that for both structures the RMSD is below 1.4 Å. This shows that both structures are practically identical to the crystallographic one, apart from the natural thermal fluctuations occurring in solution. The structural characterization is completed by computing the time evolution of other fundamental structural properties: the gyration radius (Rg), and the β−sheet and α−helix content (all shown in Figure S4.). Their mean values and the corresponding standard deviation obtained in the last 200 ns are summarized in Table 1. All in all, the obtained values not only support the structural stability of both structures but are also in agreement with previous works [16,19,66,67]. The latter confirms the validity of the force fields here used for describing the structure and dynamics of the wild-type protein.

Concerning the comparison between wild-type protein with its three mutants, i.e., K41C, L120C and S89C, similar results were obtained. As shown in Figure 2, the mutant structure is practically superimposed to the wild-type average conformation. This suggests that point-like mutations have little effect on the stability of the protein as a whole, in agreement with previous experimental findings [2]. In fact, this result could be anticipated considering the large amount of hydrogen bonds present in the Azurin molecule, e.g., the ones stabilizing the rigid β-barrel structure. Computing the RMSD between the wild-type and mutated configurations ($RMSD_{Mut}$ in Figure 2) we realize that $RMSD_{Mut}<\;$0.45 Å for the three mutations. This highlights that the difference between the mutants and the wild-type protein is even smaller than the difference between the wild-type configuration and its crystallografic structure arising from thermal fluctuations (∼1.05 Å, see Figure S4). This result can be understood in light of the position of the mutated residues, which are located at the protein surface (see Figure 1 and Figure S1). Consequently, the internal sidechain-sidechain interactions between different amino-acids remain unaltered, which helps to a better preservation of the structure of the protein as a whole [68]. Finally, the structural characterization of the mutants is completed by computing the time evolution of the RMSD, the gyration radius and the secondary structure fluctuations (see Figure S4). The average values and their standard deviations are reported in Table 1. Interestingly, regardless of the position of the mutation the observed differences between the wild-type protein and its mutants are smaller than the thermal fluctuations. Also the secondary structure analysis shows that the folding of the protein remains virtually unaltered upon the introduction of the mutation (see Table 1). This finding is consistent with previous experimental observations, where the structure of other Azurin mutants were analyzed both through their absorption spectra [69] as well through their crystallographic structure [70].

### 3.2. Dynamics/Fluctuations of Unrestrained Wild-Type and Its Mutants in Water

**Figure 3:**
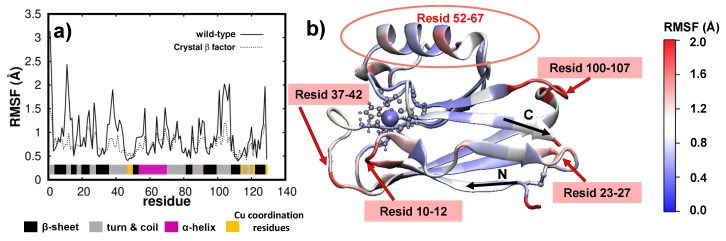
**Fluctuations (RMSF) per amino-acid of an unrestrained wild-type protein in water.** (**a**) RMSF of each wild-type residue over the last 200 ns of MD simulation. The same values obtained from the crystallographic β-factors [34] are also included with a dashed line.Secondary structure of each amino-acid is also detailed. (**b**) RMSF data for each residue/amino-acid represented directly over the wild-type protein averaged structure. The Azurin representation used is the same as in Figure 2.

The dynamics of the protein may be accessed via the time averaged fluctuations of each amino-acid composing the Azurin. The averaging was performed in the last 200 ns of simulation as then the protein structure/dynamics is in thermal equilibrium (see Figures S4 and S6). It is worth mentioning that the fluctuations per amino-acid obtained over the last 200 ns of the wild-type and K41C simulations (t = 300–500 ns) are quite similar to the ones obtained from 100 to 300 ns of simulation (see Figure S5), thus indicating that a 300 ns simulation-long suffices for a proper description of the Azurin fluctuations. The fluctuations per amino-acid for the wild-type protein (computed as the root-mean-square-fluctuations, see Methods) are shown in Figure 3a and represented on top of the protein structure in Figure 3b. Additionally, for comparison purposes in Figure 3a we also represent the RMSF values derived from the crystallographic β-factors [34]. By comparing the results of our simulations with crystallographic data two main features become apparent: firstly the location of the most flexible regions is the same for both MD and crystallographic data (note that the peaks appear in the same place in both cases); secondly the fluctuations predicted from the crystallographic data are quenched with respect to our MD simulations. Considering the amplitude of the fluctuations one must bear in mind that crystallographic data only provides an indicative of the atomic motion/disorder of the protein crystal, whereas the MD simulations also account for concerted oscillations of different parts of the protein due to the presence of the solvent and the temperature of the system [16,71]. Therefore we may argue that our simulations are able to correctly describe the fluctuations/dynamics of the wild-type protein.

Further insight into the wild-type protein fluctuations, particularly of how they are distributed over the whole molecule, is obtained through their spacial representation as shown in Figure 3b. There, we observe that residues located in *random-coils* or *turns* are generally more flexible (1.0–2.0 Å, shown in red in Figure 3b) than the rest. Alternatively, regions with well defined secondary structure held together by strong hydrogen-bond networks, such as in β−sheets, barely fluctuate (0–1.0 Å, in blue). These findings are general in nature, as similar behaviour is observed in many other proteins [32,63,64,65]. Regarding the wild-type Azurin, we observe that its largest fluctuations (>1.8 Å) are located in three different amino-acid segments, all of them belonging to turn regions: residues 10–12, residues 37–42 and residues 100–107 (see Figure 3b). This is also in agreement with previous simulations [16,17,20,72,73]. It is still worth noting that the only *turn* regions where fluctuations are hampered (i.e., <0.8 Å) are the ones near the five coordination residues of the Cu ion. This shows that the Cu-coordination promotes a rigidification of the neighbouring residues regardless of being located in a soft/hard secondary structure.

**Figure 4:**
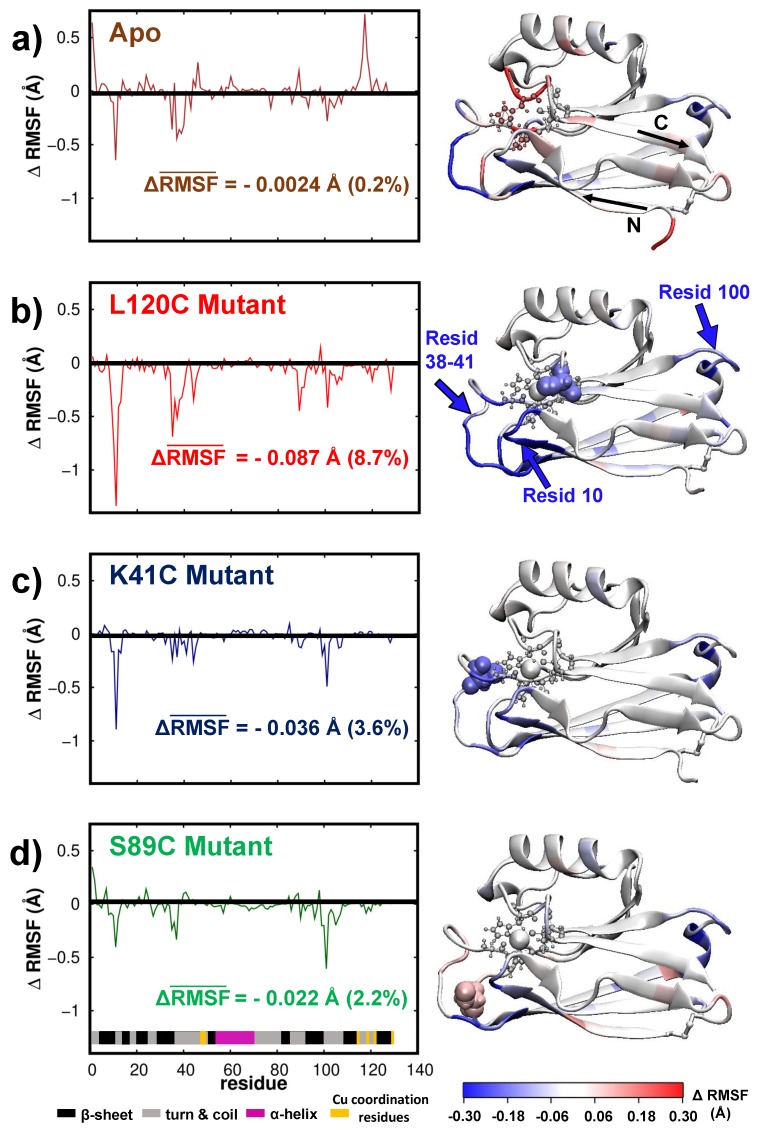
**Differential fluctuations of the Apo and mutants with respect to the wild-type protein.** (**a**–**d**) In the left panel we plot the $\Delta \;RMSF\left(i\right)=RMSF^{j}\left(i\right)-RMSF^{wild-type}\left(i\right)$, being (i) the amino-acid and (j) the different structures considered which are indicated inside the plot as text. Secondary structure of each amino-acid is detailed as in Figure 3a. On the right panel we represent with a color scale these values on-top of the protein’s structure. Please note that for each protein we also compute the difference between its mean fluctuation value with the mean fluctuation of the wild-type protein, i.e., $\Delta \;\bar{RMSF}=\frac{1}{128}\cdot \sum_{i=1}^{128}RMSF\left(i\right)-RMSF^{wild-type}\left(i\right)$.

To understand the role of the Cu-coordination on the fluctuations/dynamics of the Azurin we now compare the dynamics of the wild-type protein with the Apo. In Figure 4a we represent the difference of the RMSF (fluctuations) computed for the Apo with respect to the one computed for the wild-type protein for all amino-acids, i.e., we represent $\Delta RMSF\left(i\right)=RMSF^{Apo}\left(i\right)-RMSF^{wild-type}\left(i\right)$. As a result, negative ΔRMSF indicates that fluctuations are smaller in the Apo, and positive ΔRMSF means that Apo fluctuates more. As anticipated, the residues of the Apo located near the copper coordination site vibrate more, with an increase of up to 0.3Å (see Figure 4a). This agrees with crystallographic data [74] which showed an increase of the flexibility of the Cu binding site in the Apo structure. Remarkably, the fluctuations in some *turn* regions of the protein (residues 38–40 and 100–103) were also significantly quenched. As a result of this compensation, the mean fluctuation of the whole Apo-protein, defined as $\bar{RMSF}=\sum_{i=1}^{128}RMSF\left(i\right)$, is essentially identical to its cooper coordinated counterpart (see Figure 4a).

As with the Apo, we analyzed the role of mutations on the wild-type dynamics/fluctuation. Figure 4b–d represents the variation of the RMSF with respect to the wild-type protein (i.e., ΔRMSF(i)) of the three different mutants (K41C, S89C, L120C) for each amino-acid. Overall, we observe that the average fluctuations are systematically quenched upon the introduction of a mutation (see values of $\Delta \bar{RMSF}$ in Figure 4b–d). Interestingly, the suppression of fluctuations is localized in the three amino-acid segments which have the higher mobility in the wild-type protein, i.e.,: residues 10–12, 38–41, and 100–103, all located inside *turn* regions of the protein. Moreover, the directional character of that fluctuations over the first of these amino-acid segments (residues 10–12) can change from a breathing to a shearing displacement in the β−barrel plane with the introduction of the mutations (see principal component analysis of these fluctuations in Figure S8). From all these results, three important conclusions may be derived concerning the role of mutations in the dynamics of a wild-type protein. First, the introduction of a mutation near the copper(II) ion can affect the dynamics of residues located elsewhere. Interestingly, this long-range effect is in agreement with previous observations [20]. Second, mutations seem to quench the largest fluctuations of the wild-type protein regardless of the position of the mutation, i.e., their proximity to those regions. Third, the creation of single amino-acid mutations can reduce the mean fluctuation of the whole wild-type protein up to a 8%, see Figure 4b–d, thus systematically promoting a stiffening of the Azurin. Here it is important to put this observation in a broader context. In many other proteins, e.g., viral capsides, it was shown that single-point mutations that preserve the protein structure always result in its stiffening [21,22]. It was suggested that this might have some evolutionary origins, as a stiffening of a protein structure may result in a reduction of its biological activity and therefore a natural selection of more mobile proteins [22]. Thus, the results obtained for the Azurin, i.e., systematic stiffening upon the introduction of mutations, although surprising, are in line with observations on other similar biomolecules [21,22]. At last, it is interesting to note that among the three mutations here considered (K41C, S89C, L120C), the largest stiffening is obtained when the mutation is closer to the copper(II) ion, i.e., the L120C structure. In fact, our results indicate that the smaller the Cu-mutation distance is, the more pronounced the effect of that mutation is on the Azurin dynamics, see Figure 1 and Figure 4b–d. This effect may be understood in-light of the strong coordination of the amino-acids surrounding the Cu site. Whereas in a standard fragment of the protein, a given amino-acid is connect to only two other, around the copper coordination site the amino-acids are connected also with other regions of the protein through the strong metal coordination bonds. Therefore, quenching the fluctuations near this site would have an impact on a larger area of the protein as compared to other not so strongly coordinated region (as is the case of mutant S89C).

### 3.3. Structure/Conformation of Wild-Type and K41C Mutant upon Adsorption to Au(111)

Henceforth we focus on the structure and dynamics of the as adsorbed wild-type and K41C mutant proteins to Au(111) surface. The choice of these proteins is motivated by recent experiments [2] which showed that this particular single point mutation drastically altered Azurin conductance. The initial protein-surface distance is larger than ∼1 nm so the protein is able to reorient. Furthermore, four different initial orientations were considered (see the motivation of those in the Methods section) so to inspect their influence on the final adsorption configuration. Please note that to best mimic experimental conditions [2], both the protein and surface are fully embedded in water (see Methods for further details).

**Figure 5:**
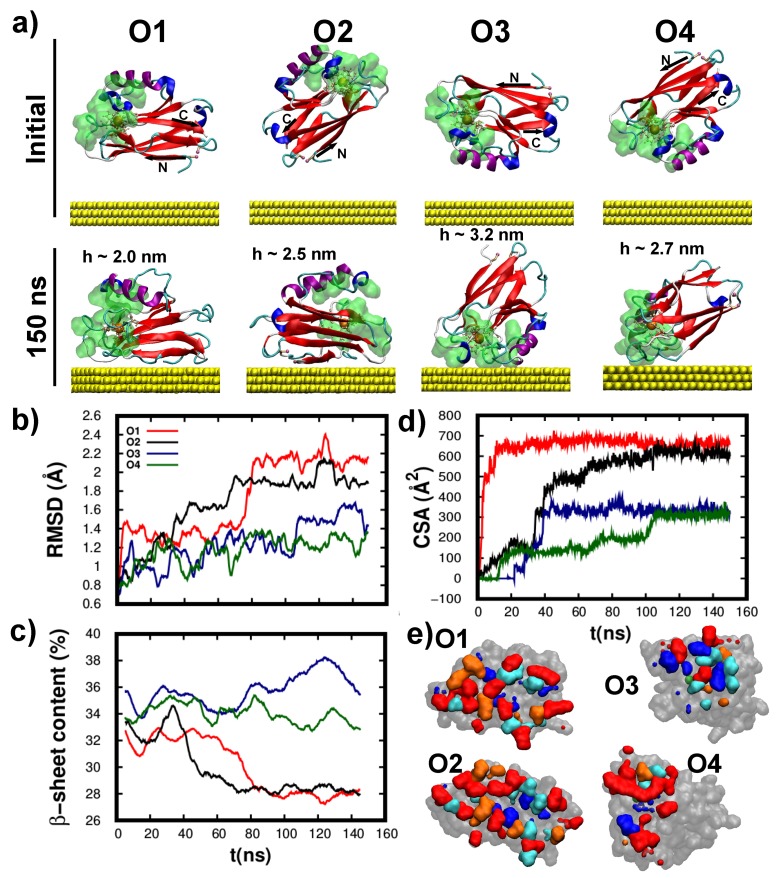
**Structural characterization of the wild-type protein adsorption over Au(111).** (**a**) Snapshots of the initial (top row) and final (bottom row) configurations of the adsorption process. The four different initial adsorption configurations are labeled as O1, O2, O3 and O4. The molecular height (h) of each final configuration is also indicated. The representation used is the analogous to Figure S7. The Azurin hydrophobic patch is highlighted with a green Connolly surface [61]. The Au atoms are represented with yellow Van-der-Waals spheres. (**b**–**d**) Time evolution of the (**b**) RMSD, (**c**) β-sheet content and (**d**) CSA for the four initial orientations considered. (**e**) Connolly surface [61] of the Azurin amino-acids directly in contact with the Au(111) after the adsorption (t = 150 ns). The amino-acids are colored according to their hydrophobicity index: very hydrophobic (blue), hydrophobic (cyan), neutral (orange), and hydrophilic (red). The Connolly surface of the rest of the Azurin residues (non-interacting) is also shown in grey (see Figure S10).

For all starting orientations (shown in Figure 5a) the wild-type protein readily adsorbed over the Au(111) slab (<60 ns), in accordance with the fast sample preparation times [2]. Remarkably, as shown in Figure 5a, the protein structure remained almost unaltered upon its adsorption. This is surprising considering that most proteins undergo significant structural rearrangements upon adsorption [32,63,64,65], a process which is more severe for smaller proteins [75] such as the Azurin. This highlights a key feature of wild-type protein for its incorporation in solid-state devices, i.e., its strong structural resilience which ultimately may allow better preserving its activity even in harsh environments. The structural stability may be quantified at the tertiary level via the RMSD (shown in Figure 5b) and at the secondary level through the β-sheet content (shown in Figure 5c). By comparing these results with the data for protein in solution (see Figure S4), we quantify the small structural change induced by the adsorption (Δ RMSD < 1Å; Δ β-sheet content < 7%). Moreover, a detailed inspection of the final adsorption configurations revealed that these structural changes are mostly located in the amino-acids sitting at the protein-surface interface (see Figure S9). In fact, the larger the contact-surface-areas (CSA – shown in Figure 5d) the larger the change in RMSD as well as in the β-sheet content. At last, we analyze the hydrophobic character of the amino-acids closer to the surface at the end of the adsorption, see Figure 5e and Figure S10. This analysis highlights the importance of the hydrophilic amino-acids in the protein-surface interaction (colored in red), as they represent more than 25% of the total contacting residues regardless of the adsorption configuration. This result was also observed in other proteins [64] and provides evidence for the larger accessibility of these residues to the surface as they are normally solvent-exposed.

The free adsorption simulations shown in Figure 5 points out that wild-type protein does not necessarily adsorb through its cysteine groups, see O3 in Figure 5. To rationalize this finding, other factors known to govern protein adsorption [32] must be considered. On the one hand, the strong short-range cysteine–Au interaction is efficiently screened by the many water layers between the protein and surface prior to its adsorption. On the other hand, once the protein-surface contact is reached, the large contact area gives rise to a significant long range non-specific Van-der-Waals interaction [32]. As shown in our simulations, the former is sufficiently large to stabilize an adsorption configuration that is not mediated by the cysteine groups. In fact, a close inspection of the adsorption configurations shown in Figure 5a reveals that wild-type protein preferentially adsorbs along two different orientations: lying-down over the substrate (O1 and O2—with two cysteines anchored to Au(111)) and a partially standing up configuration (O3 and O4—tethered to the surface via the hydrophobic patch). Interestingly, this qualitative assessment is also observed in the quantitative structural analysis as all figures of merit (RMSD, β−sheet content and CSA) show two distinct trends. In particular, the lying-down orientation gives rise to a systematically larger CSA (see Figure 5d,e) which in turn results in a larger change of both the tertiary (see Figure 5b) and secondary (see Figure 5c) structures. The two adsorption orientations also result in different molecular heights (h), with h∼2.25 nm for the lying-down and h>2.6 nm for the partially standing up configuration (see Figure 5a). This is in agreement with previous MD simulations [19] and Atomic-Force-Microscopy measurements [76,77], which also predicted a high variability in the dimensions of the Azurin adsorbed over Au(111). This high variability may be important to understand Azurin junctions conductance experiments, as the protein-electrodes coupling highly depends on the protein orientation [18]. At last, it is interesting to note that for some initial configurations, the protein undergoes a major reorientation process to finally adsorb either through the cysteines or the hydrophobic patch. This is especially notorious for the O3, where the protein rotates over ninety degrees so to finally adsorb via the hydrophobic patch.

**Figure 6:**
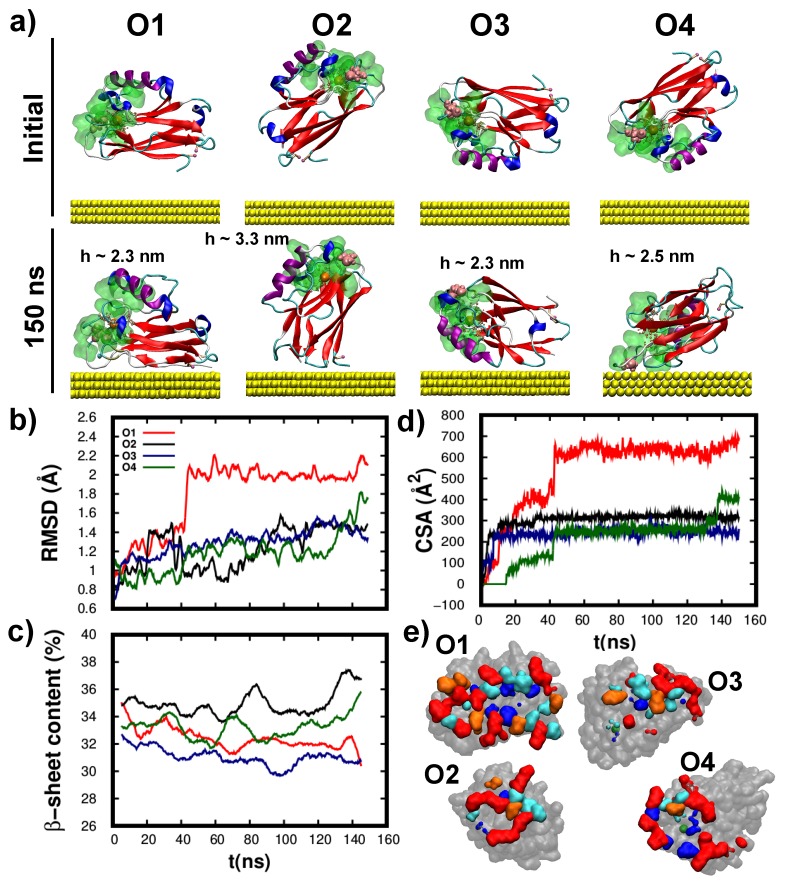
**Structural characterization of the K41C adsorption over Au(111).** (**a**) Snapshots of the initial (top row) and final (bottom row) configurations of the adsorption process. The representation and the initial orientations considered are identical to the wild-type case shown in Figure 5. The mutation site is represented with pink Van-der-Waals spheres. The molecular height (h) of each final configuration is shown. (**b**–**d**) Time evolution of the (**b**) RMSD, (**c**) β-sheet content and (**d**) CSA for the four initial orientations considered. (**e**) K41C amino-acids directly in contact with Au(111) after the adsorption. The representation is identical to Figure 5e (see Figure S10).

The K41C adsorption simulations, see Figure 6a, revealed that it readily adsorbed onto the Au(111) surface. As with the wild-type protein, K41C adsorption resulted in minor structural rearrangements of both the tertiary (see Figure 6b) and the secondary (see Figure 6c) structures. Here again, the larger the protein-substrate contact area (i.e., O1) the more pronounced were the structural rearrangements (notably the RMSD shown in Figure 6b), thus supporting that they are mostly located at the interface. Interestingly, the additional K41C cysteine did not promote cysteine-tethered configurations. This observation supports that the Azurin adsorption process is governed by the detailed balance of many different interactions/processes as aforementioned. Lastly, the as adsorbed K41C molecular heights (h—shown in Figure 6a) also showed a large dispersion consistent with the results obtained for the wild-type protein.

In the wild-type protein the adsorption proceeded either through the hydrophobic patch or via the cysteine groups, interestingly this is not the case for the K41C as each starting orientation gives rise to a different adsorption configuration, see Figure 6a. These may be structurally described as: lying-down with the cysteines in contact with the surface (O1), lying-down with the cysteines far from the surface (O3), upright tethered via the cysteine groups (O2) and upright anchored via the hydrophobic patch (O4). As a result, the final CSA values (see Figure 6d,e) are different for each starting configuration. Interestingly, the two K41C adsorption configurations which were similar to the wild-type case, i.e., lying-down with the cysteines tethered to the surface (O1) and the one anchored via the hydrophobic patch (O4), have a tertiary/secondary structure (RMSD/β−sheet cont.) very similar with the ones obtained for the wild-type protein. Despite the undisputed merit of these and other protein adsorption simulations [32,64,65], they all rely on the fundamental assumption that a thermal equilibrium was reached. It is extremely difficult, if not impossible, to determine, from a theoretical study alone, if a MD simulation has reached an actual equilibrium configuration and therefore we do not exclude that other adsorption orientations might coexist as stable/meta-stable states. Possible routes to explore this include: performing longer simulations, additional starting orientations or via the use of accelerated sampling methods. Nevertheless none would provide a ultimate proof of having reached an actual thermal equilibrium state which can only be provided through high resolution experiments [65] that thus far has remained elusive for Azurin on gold. Concerning K41C adsorption, we observe that in the four cases here considered the protein does not reorient itself, i.e., it adsorbs along its initial orientation, in contrast with our findings for the wild-type protein. Notably, starting from the O2 orientation, the wild-type protein is able to reorient itself to adsorb lying down over the substrate, whereas K41C barely changes its orientation as it adsorbs vertically. A similar lack of mobility is observed for the O3 case. Although our simulations do not provide a direct proof of this effect, still they point into the direction that quenching protein dynamics/fluctuations via point-mutations (see Figure 4) might have implications on its adsorption process, even prompting to the variation of the most statistically probable adsorption configurations. This variation of the protein-electrode configuration would influence the electrode-protein coupling [18], which could ultimately affect to the conductance ability of this protein on the junction [5]. Our hypothesis linking the stiffening of the protein upon mutations and the changes in the adsorption behavior—which we hope that will stimulate future work–, is supported by recent findings showing protein adsorption is ultimately guided by entropic effects at the protein-solvent-surface interface [32]. Quenching fluctuations at a given region of the protein will necessarily hamper its ability to push away the water molecules at the interface via thermal fluctuations and restricts its ability to change the conformation of the amino-acids at the protein-surface interface required to achieve a stable adsorption configuration.

### 3.4. Dynamics/Fluctuations of Wild-Type and K41C Mutant upon Adsorption to Au(111)

**Figure 7:**
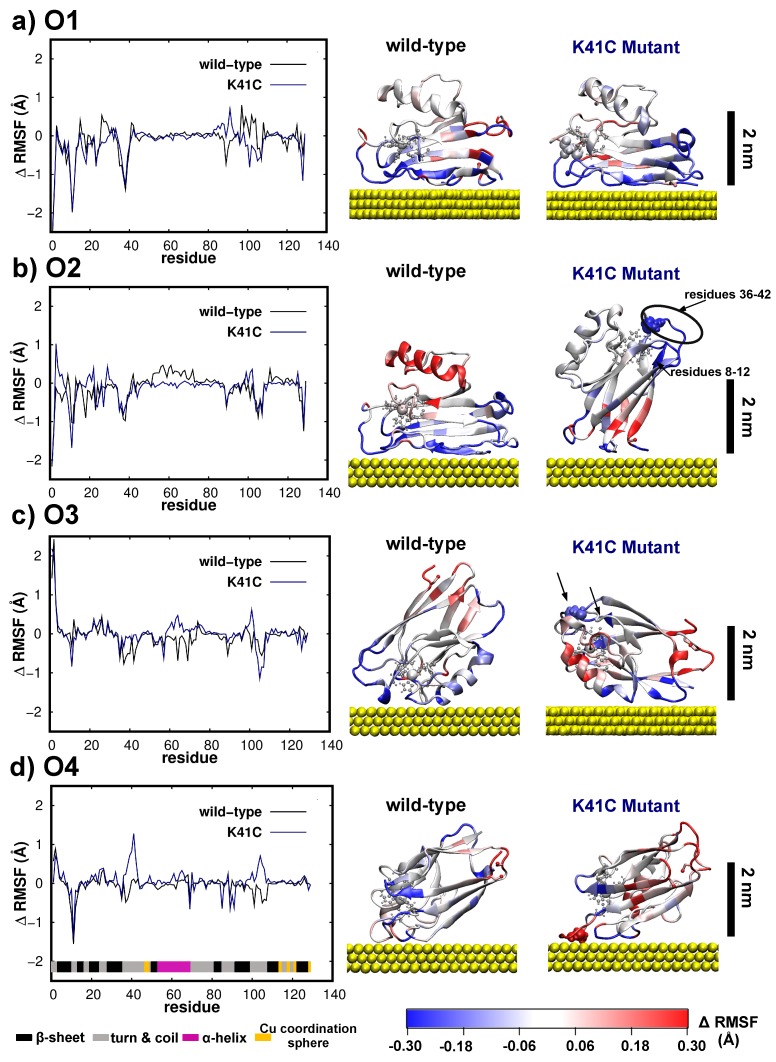
**Differential fluctuations of the wild-type and K41C proteins adsorbed with respect to wild-type protein in solution.** Here we show the $\Delta RMSF=RMSF^{adsorbed}-RMSF^{wild-type,free}$ averaged from the last 70 ns of simulations for the four initial orientations (**a**) O1, (**b**) O2, (**c**) O3 and (**d**) O4. On the left column in black/blue we represent the ΔRMSF value of the wild-type/K41C per amino-acid. The secondary structure character of each amino-acid when the wild-type protein is free solved in water is also specified (see Figure 4). In the right column we show ΔRMSF projected in the equilibrium adorption configuration. The position of the K41C mutation is highlighted with a van-der Waals representation. The Azurin color representation is the same as in Figure 4.

Further insights into the dynamics of the as-adsorbed proteins are provided through the difference between its thermal fluctuations (RMSF) after the adsorption and the RMSF of the wild-type protein in solution, i.e., $\Delta RMSF=RMSF^{adsorbed}-RMSF^{wild-type,free}$. The differential fluctuations per residue over the last 70 ns of simulations are shown in Figure 7 for the four different starting configurations of the two proteins here considered, i.e., wild-type and K41C proteins. Overall, the adsorption process impacts the protein fluctuations/dynamics in three well defined regions: near the surface, far away from it and in the three regions of the Azurin with intrinsic enhanced mobility [16,17,20,72,73] highlighted in Figure 3. The amino-acids in direct contact with the surface are naturally restrained due to the adsorption process, thus showing much smaller fluctuations that they would have in the liquid phase (i.e., $\Delta RMSF=RMSF^{adsorbed}-RMSF^{wild-type,free}<0$, blueish regions in Figure 7). On the other hand, the residues located far from the surface display an enhanced mobility as shown by their red coloring (ΔRMSF>0) in the second column of Figure 7. The mechanism behind such enhanced mobility results from the fact that the other end of the protein tethered to the surface works as hinge transferring part of the thermal fluctuation energy into global motion most noticed at regions located far from the surface. At last, the amino-acids located in highly flexible regions (residues 10–12, 37–42 and 100–107) experience significant changes in their dynamical properties as a result of the adsorption process. Although small discrepancies among the different orientations, in all cases this effect is felt regardless of the position of these regions with respect to the surface (see first column of Figure 7). Perhaps the most interesting case, since its behavior deviates from the rest, is the O4 orientation for K41C. There, we observe that intrinsically mobile regions, especially the second segment which contains the mutation, experience an enhancement of their fluctuations (corresponding to the positive peaks in Figure 7d) thus deviating from the general trend of quenching fluctuations in these regions as a result of the adsorption. The first aforementioned enhanced mobility peak may be understood in light of a major rearrangement of the Cu coordination sphere arising from the adsorption of the mutated cysteine onto Au(111).

**Figure 8:**
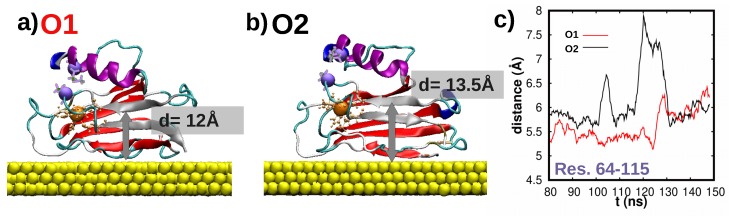
**Structural differences between O1 and O2 equilibrium adsorption configurations for wild-type Azurin.** Final snapshots obtained for the (**a**) O1 and (**b**) O2 orientations. The representation used is the same as in Figure 5. The β−sheets near the α−helix (S7 and S8 β−sheets, see Figure S9) [16] are highlighted in gray. The distance between these β−sheets and the Au(111) surface is also shown. The distance between the Cu center and the end atom of the α−helix, both highlighted via a Van-der-Waals sphere in both (**a**,**b**), is represented in the plot shown in (**c**) for both orientations, i.e., O1/O2 in red/black.

Surprisingly, Figure 7a,b shows that *equivalent* adsorption configurations of the wild-type protein may result in different dynamical behavior. This is particularly notorious in the α-helix region, which remained stationary/mobile in the O1/O2 adsorption configuration. Such difference will certainly affect the stability of molecular contacts especially in ’blinking’ experiments [2], where contact may be reached further away from the surface and with a higher frequency when the α-helix exhibits a mobile character, i.e., moving closer and away from the surface such as in O2 case. As shown in Figure 8a for O1, the β−sheets nearer to the α−helix (S7 and S8 β−sheets) [16] are more strongly adsorbed to the Au(111) which then restrains the α-helix motion (shown in Figure 8c). Contrastingly, in the O2 orientation a slight roll of the β-barrel equilibrium configuration over the surface gives rise to a larger distance between the surface and the S7 β−sheet, the S8 β−sheet and the α−helix. This in turn translates into a smaller interaction of these sites with the surface, see Figure 8b,c, which ultimately results in a much larger mobility of the the α−helix. All in all, this highlights the interplay between fine structural detail of the as-adsorbed protein and its dynamical properties which will certainly affect the stability of biomolecular contacts.

Concerning the mobility of K41C, we now focus on the orientations which showed an overall decreased mobility as compared to the wild-type protein, i.e., O2 and O3, as they are unable to reorient during the adsorption process (see Figure 6). In Figure 7 we observe that in both orientations the regions located near the mutation site (residue 41) experience a strong quenching of their fluctuations, or equivalently are significantly stiffer than the corresponding wild-type protein segments (colored in blue—ΔRMSF<0). Interestingly, in both cases this region is located far away from the surface, i.e., at a distance larger than 2 nm, pointing out that this quenching of fluctuation is not related to the adsorption process but is instead a direct consequence of the mutation. Since the protein adsorption process is essentially driven by entropic forces and Van-der-Waals forces (directly dependent on the contact area) [32] it is sensible to assume that soft regions which are able to accommodate larger deformations as a result of the adsorption process will be favored over rigid ones. Therefore, these results seem to suggest that single-point mutations not only affect the overall protein mobility and stiffness but this in turn may also affect equilibrium adsorption configurations. Although our simulations do not provide a direct validation of this effect, we hope that our results might stimulate future works, such as high-resolution AFM images of Azurin on gold, to conclusively prove/disprove this hypothesis.

## 4. Conclusions

In this work, we firstly studied how single-point mutations (K41C, L120C, S89C) affect the structure and dynamics of the wild-type protein in solution via all atom MD simulations. The mutations here considered barely changed the wild-type protein. Interestingly, they systematically result in a stiffening of the structure as a whole. Although similar findings were already reported in other biomolecules [21,22], this effect was not previously reported/considered in the Azurin. Such modification of the protein dynamical/mechanical properties certainly plays a relevant role in their stability in solid-state junctions. In the second part of this work, we considered the role of such mutations in the structure and dynamical properties upon their adsorption to a Au(111) surface. Our adsorption simulations showed that the protein structure remained almost unaltered upon its adsorption. Such structural resilience differentiates Azurin from the most common blood plasma proteins [32,63,64,65] where larger changes in both tertiary and secondary structures are expected, especially considering the small size of this protein. Also, our free adsorption simulations revealed a surprisingly high mobility of the wild-type protein which was able to undergo major reorientation maneuver during the adsorption process. Interestingly, such mobility allowed the wild-type protein to systematically adsorb along one of two favored conformations: either with the hydrophobic patch facing the Au(111) surface or tethered by the cysteine groups to the surface. However, for the K41C mutant, our four different adsorptions simulations showed that the Azurin was unable to reorient itself. This resulted in a change of the preferred Azurin configurations leading to the emergence of other two possible adsorption configurations (standing up with the cysteines in contact with the surface, lying-down with the cysteines far from the surface). Interestingly, a detailed comparison between the fluctuations of the free wild-type Azurin and all the mutants (in particular K41C) unveiled a clear stiffening effect induced by the mutations. Given the importance of thermal fluctuations in the adsorption process [32], here we hypothesize that quenching them through the introduction of mutations might ultimately affect how the Azurin adsorbs over Au(111). Although direct validation of this hypothesis remained elusive, we hope that the indirect evidence here provided might motivate future works. These results shed light on how two fundamental properties (structure and dynamics) of biomolecular contacts may be tuned via single-point mutations, both known to have major implications in the electron-transport properties of these contacts [18].

## Figures and Tables

**Table 1:** Mean and standard deviation values of the: RMSD, Rg, α-helix and β-sheet content in the last 200 ns of MD simulations.

	RMSD (Å)		Rg (Å)		α-Helix Content (%)		β-Sheet Content (%)	
	Mean	$\sigma_{t}$	Mean	$\sigma_{t}$	Mean	$\sigma_{t}$	Mean	$\sigma_{t}$
**wild-type**	1.086	0.158	14.049	0.051	10.156	1.893	36.149	1.601
**Apo**	1.099	0.137	14.061	0.062	11.151	2.128	35.299	2.002
**K41C**	1.021	0.121	14.051	0.021	10.912	1.952	35.916	1.722
**L120C**	0.980	0.107	14.036	0.065	11.049	1.986	34.446	1.491
**S89C**	1.100	0.110	14.062	0.060	9.319	1.532	35.674	1.463

## Supplementary Materials

The following are available online at https://www.mdpi.com/2218-273X/9/10/611/s1; Figure S1: Side and top views of the initial configurations of the Azurin; Figure S2: Cu-mutation distance distributions obtained from the unrestrained Azurin simulations in water; Figure S3: Evolution of the minimum distance between the Azurin protein and its periodic image during all the MD simulations performed in this work; Figure S4: Structural characterization of the five unrestrained Azurin structures in water; Figure S5: Time-dependence of the protein root-mean-square fluctuation (RMSF) during its unrestrained simulation in water; Figure S6: Evolution of the energy of the system during the unrestrained Azurin MD simulations in water; Figure S7: Characteristics of the MD simulations of the Azurin adsorption process; Figure S8: Principal component analysis for the Apo, wild-type and K41C proteins during their unrestrained dynamics in water; Figure S9: Characterization of the wild-type regions where the β-sheet content is lost during the adsorption simulation; Figure S10: Hydrophobic level description of the protein-surface contacting areas. Supplementary Movie S1: First PCA component of the wild-type Azurin unrestrained simulation in water highlighting vibrations in the cooper coordination sphere; Supplementary Movie S2: First PCA component of the Mutant K41C unrestrained simulation in water highlighting vibrations in the cooper coordination sphere; The pdbs of the four initial wild-type Azurin orientations used for studying the adsorption process of this protein on a gold surface are also provided.

## Abbreviations

The following abbreviations are used in this manuscript:

MD	Molecular Dynamics
CSA	Contact Surface Area
Rg	Radius of gyration
RMSD	Root Mean Square Displacement
RMSF	Root Mean Square Fluctuations
