# Supplementary material for: Tuning Structure and Dynamics of Blue Copper Azurin Junctions via Single Amino-Acid Mutations
Source: Biomolecules. 2019 Oct 15;9(10):611. doi: 10.3390/biom9100611 (PMC6843909; doi:10.3390/biom9100611)
Supplement: Supplementary file 1 [file biomolecules-09-00611-s001.zip › Supplementary File/Azurina_SI.pdf]

# Supporting Material for: Tuning Structure and Dynamics of Blue Copper Azurin Junctions via Single-Amino-Acid Mutations.

Maria Ortega,[†] J. G. Vilhena,[*,†,‡] Linda A. Zotti,[†,¶] Ismael Díez-Pérez,[§] Juan Carlos Cuevas,[†,¶] and Rubén Pérez[*,†,¶]

†*Departamento de Física Teórica de la Materia Condensada, Universidad Autónoma de Madrid, E-28049 Madrid, Spain*

‡*Department of Physics, University of Basel, Klingelbergstrasse 82, CH-4056 Basel, Switzerland*

¶*Condensed Matter Physics Center (IFIMAC), Universidad Autónoma de Madrid, E-28049 Madrid, Spain*

§*Department of Chemistry, Faculty of Natural & Mathematical Sciences, Kings College London, Britannia House, 7 Trinity Street, London SE1 1DB, UK*

E-mail: guilhermevilhena@gmail.com; ruben.perez@uam.es

October 2, 2019

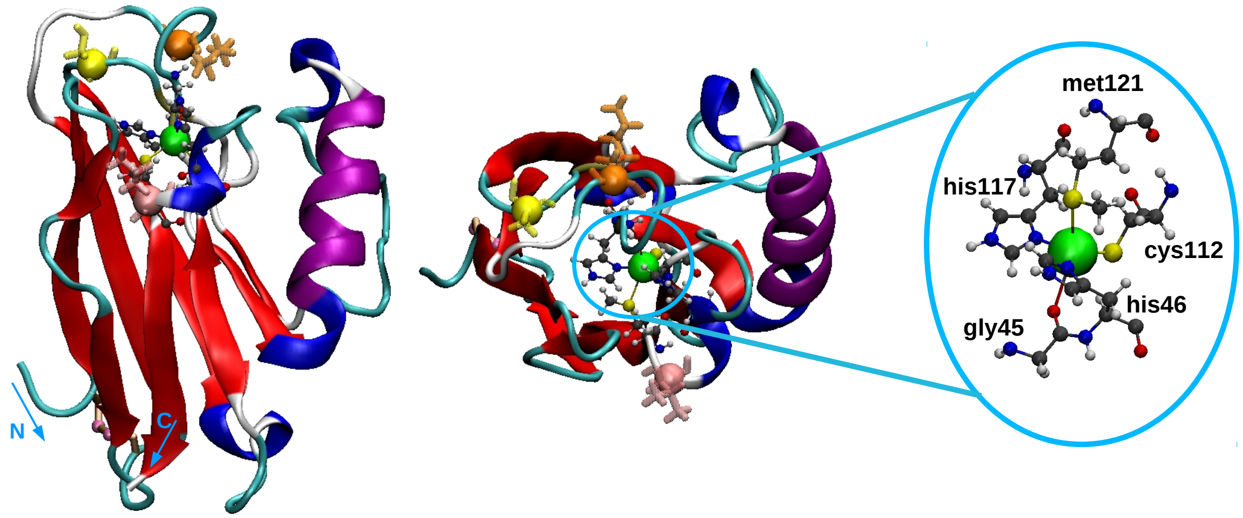


Figure S1: **Side and top views of the initial configurations of the Azurin.** The Azurin representation used is analogous to the one shown in Fig. 1 of the main manuscript. The position of the residues in which the mutations have been performed (K41,L120 and S89C) has been marked with a van-der-Waals representation in an opaque color (orange,pink and yellow respectively). A detailed view of the Cu complex of the protein, i.e the five residues of the Cu coordination sphere is shown in the inset.

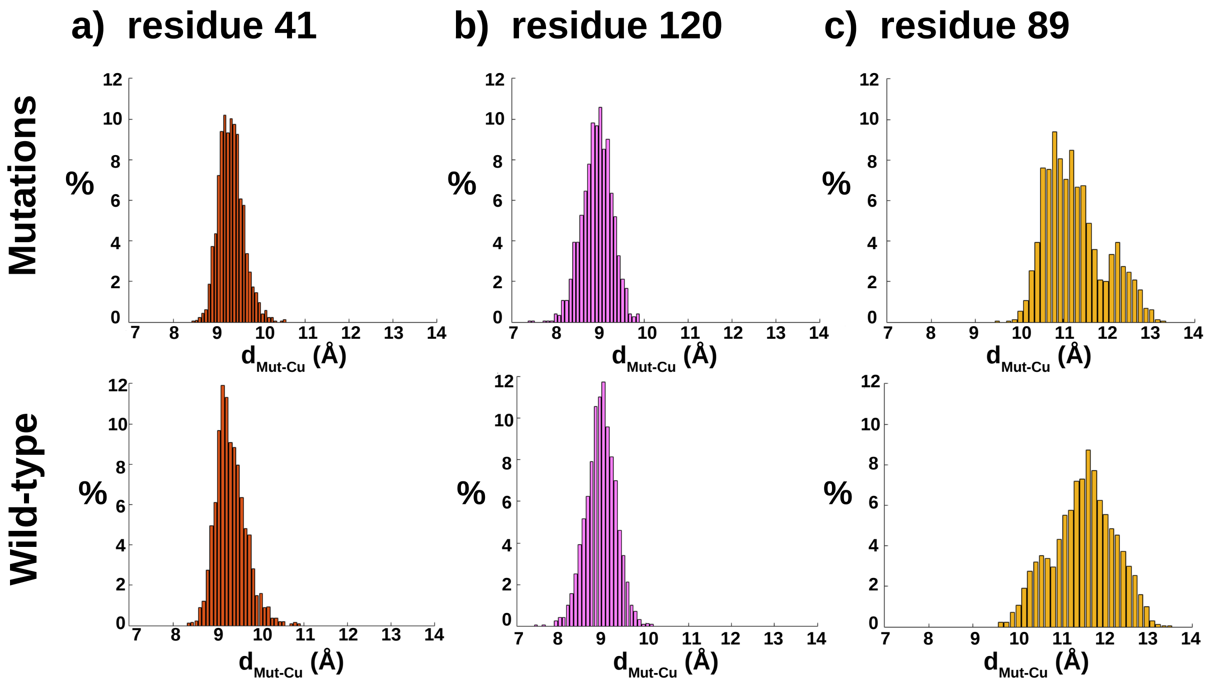


Figure S2: **Cu-mutation distance distributions obtained from the unrestrained Azurin simulations in water.** Probability of occurrence (%) of a given $d_{Mut-Cu}$ value when **a)**Mut=residue 45 (lysine), **b)**Mut=residue 120 (leucine) and **c)**Mut=residue 89 (serine). The same distance distributions obtained when that amino-acids are not mutated, i.e. in the wild-type protein, are also shown for comparison (bottom panel). The definition of $d_{Mut-Cu}$ is included in the caption of Fig.1 of the main manuscript.

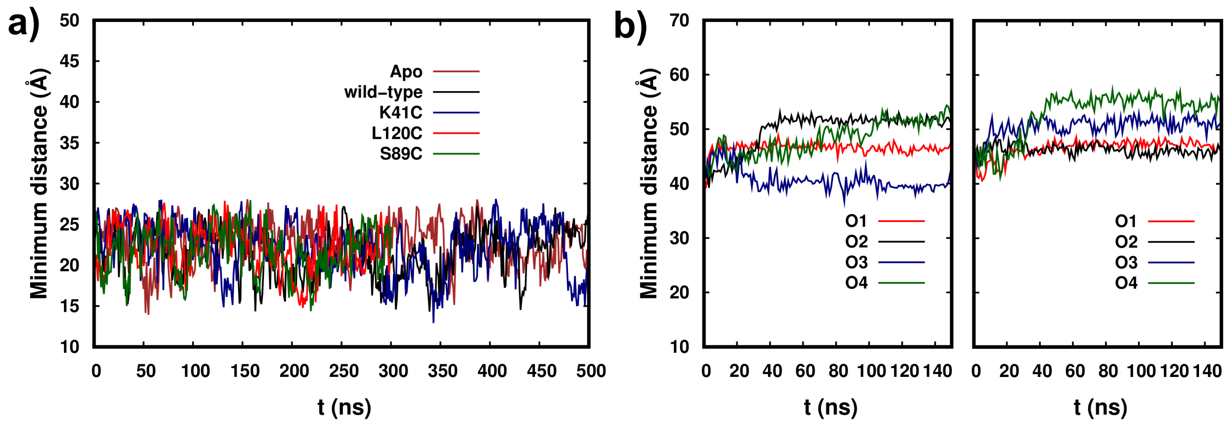


Figure S3: **Evolution of the minimum distance between the Azurin protein and its periodic image during a)the unrestrained MD simulations in water;b)the adsorption MD simulations.** For all the simulations, the minimum distance between the protein and its periodic images is larger than the value of the real-space cut-off used in these simulations (10Å), evidencing that the Azurin does not interact with its periodic images in any of them.

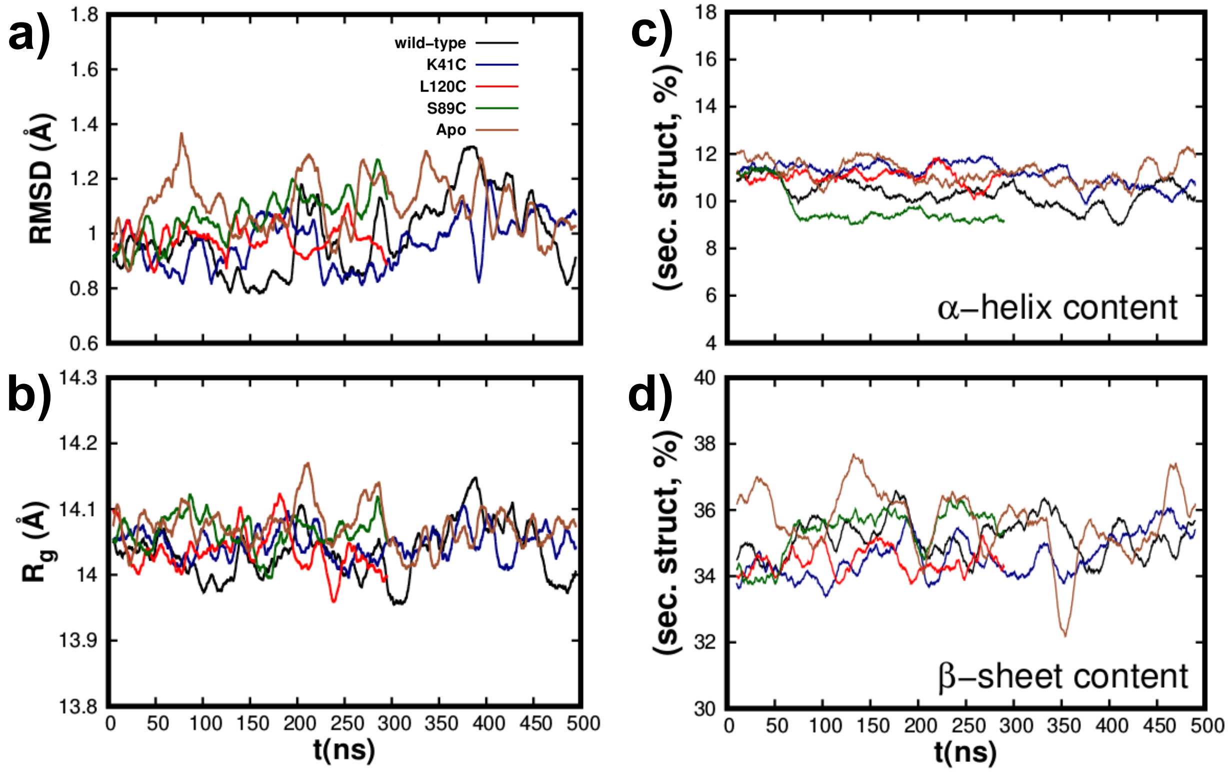


Figure S4: **Structural characterization of the five unrestrained Azurin structures in water.** Time evolution of the **a)**RMSD, **b)**the Rg, and the percentage of **c)**$\alpha$-helix and **d)**$\beta$-sheet content for all proteins considered. The reference configuration used for computing the RMSD evolution is the crystallographic Azurin structure. Note that the simulations of the L120C and S89C were stopped earlier (at 300 ns) as they were already converged at this time.

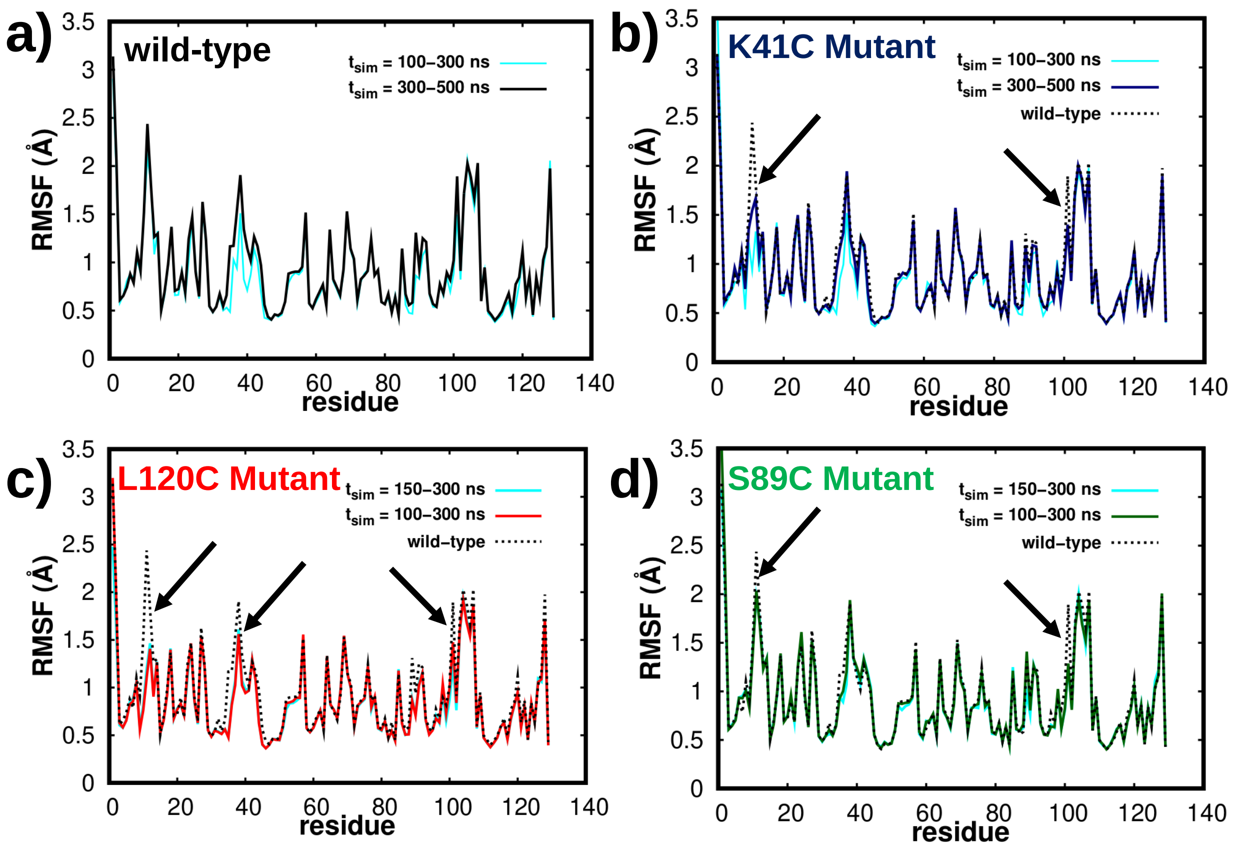


Figure S5: **Time-dependence of the protein root-mean-square fluctuation (RMSF) during its unrestrained simulation in water. a-b)** RMSF per residue calculated in the time range $t_{sim}$=100-300 ns of the **a)**wild-type and **b)**K41C unrestrained simulations in water. It is represented with a continuous cyan line. The value of the RMSF obtained using the last 200ns of these two simulations ($t_{sim}$=300-500 ns) is also here shown for comparison. As observed in these figures, the RMSF distribution along the protein is practically identical in these two time ranges and for both Azurin structures, thus indicating that 300 ns of simulation are enough for a proper description of the unrestrained Azurin fluctuations in water. **c-d)** RMSF of each residue over the last 150 ns ($t_{sim}$=150-300ns) of the **c)**L120C and **d)**S89C unrestrained simulations in water (cyan).The value of the RMSF obtained using the last 200ns of these two simulations ($t_{sim}$=100-300ns) is also here shown for comparison. From these figures, we observe that the suppression of the wild-type fluctuations (dashed line) with the introduction of mutations is even detected using a time range sma (150 ns) than the one used for the RMSF data displayed in the main manuscript (200 ns). The black arrows point to the residues where this suppression effect is larger ($RMSF_{wild-type} >> RMSF_{Mutant}$), i.e. the amino-acid segments where larger RMSF differences are detected between the Mutant and the wild-type ($|\Delta\ RMSF| > 0.3$Å, see Fig.4 of the main manuscript).

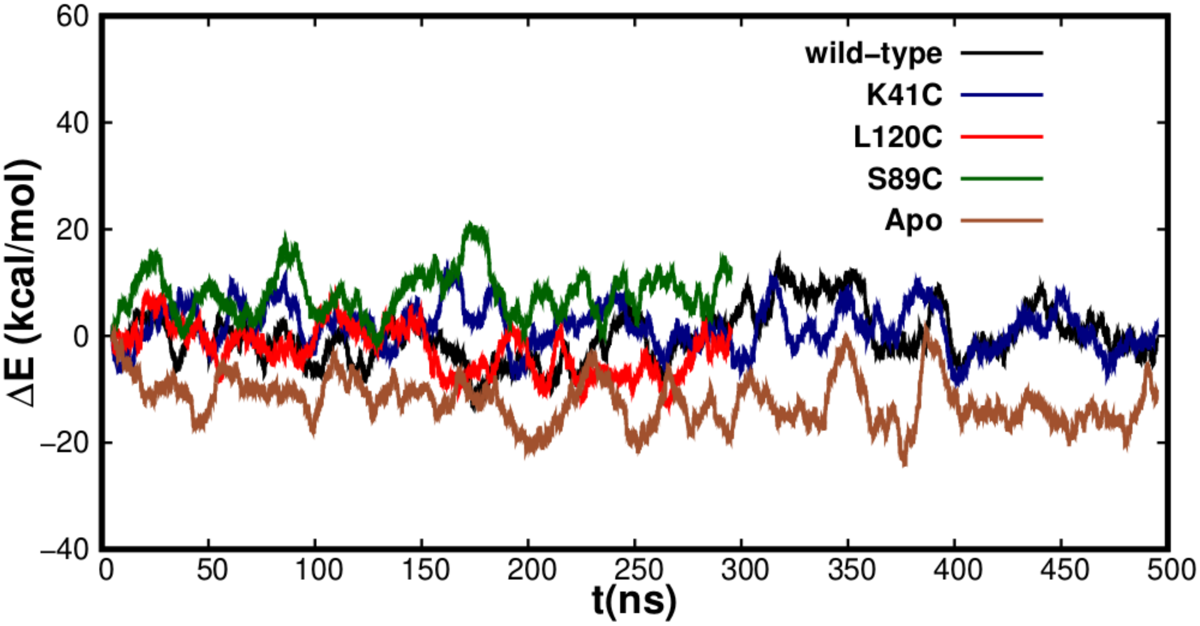


Figure S6: **Evolution of the energy of the system during the unrestrained Azurin MD simulations in water.** We show the energy evolution obtained for the wild-type (black), K41C mutant (blue), L120C mutant (red), S89C mutant (green) and Apo (brown) proteins. The average of the total energy in the first 10 ns has been taken as our energy reference. The L120C and S89C results go only until 300 ns as these simulations are shorter than the other three.

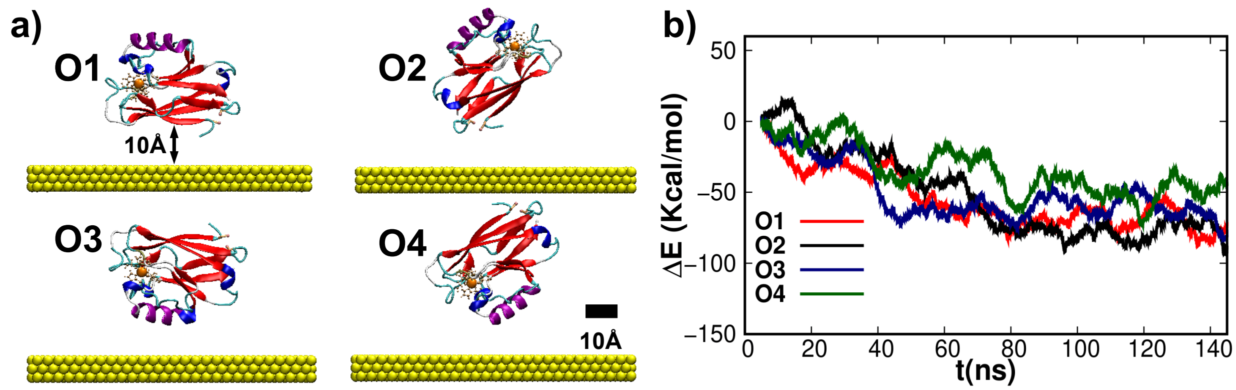


Figure S7: **Characteristics of the MD simulations of the Azurin adsorption process. a)**Representation of the four initial orientations of the wild-type over the whole gold surface considered in this work: O1, O2, O3 and O4. The protein is represented with its secondary structure as in Fig. 1 of the main manuscript. The copper atom is shown using its van-der-Waals representation in an opaque orange color, and its coordination residues are also represented in orange with ball-stick model. The main chains of the two cysteines are colored in light orange and their sulfur atoms are highlight in pink. The gold atoms are represented with a van-der-Waals representation in an opaque yellow color. **b)**Time evolution of the total energy variation during the simulation of the wild-type adsorption process for each initial orientation: O1 (red), O2 (black), O3 (blue) and O4(green). The average of the total energy in the first 10 ns has been taken as our energy reference.

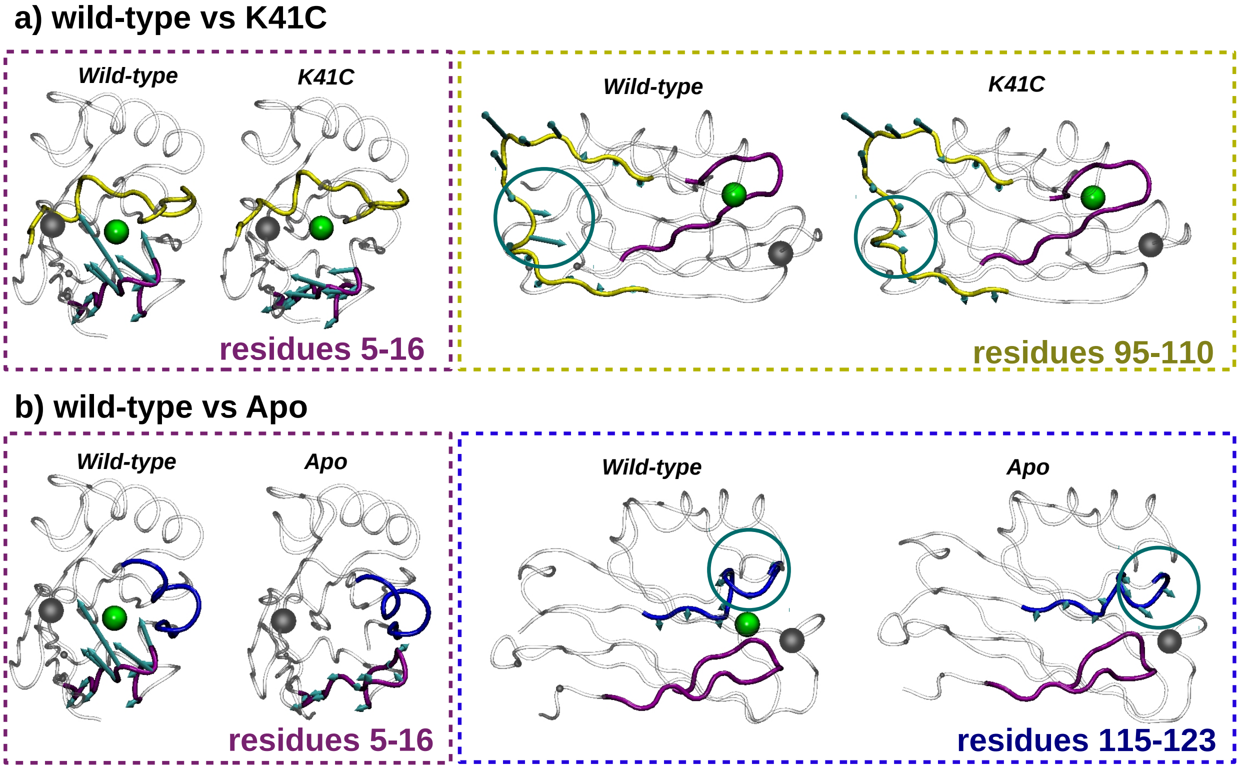


Figure S8: **Principal component analysis for the Apo, wild-type and K41C proteins during their unrestrained dynamics in water (last 200ns of simulation).** Here we illustrate the principal component direction (arrows) for the two amino-acids segments where larger RMSF differences ($|\Delta\ RMSF| > 0.5$Å) are observed between **a)** the wild-type and K41C mutant proteins; **b)** the wild-type and Apo proteins (see Fig. 4 of the main manuscript). In one of these amino-acid segments (residues 5-16), we notice not only that the fluctuations are smaller with the introduction of the K41C mutation, but also that they change its directional character from a breathing movement (wild-type) to a shearing displacement in the direction parallel to the $\beta$-barrel (K41C Mutant, see movies for more details). This shearing displacement observed in the 5-16 amino-acid segment with the introduction of the K41C mutation is also observed for the Apo. In the other amino-acid segment, i.e. residues 95-110/115-123 for the Mutant/Apo case, we observe smaller/larger fluctuations amplitude than in the wild-type (see regions marked with a circle), as predicted by the RMSF results (Fig. 4 of the main manuscript), but the directional character of the fluctuations is the same.

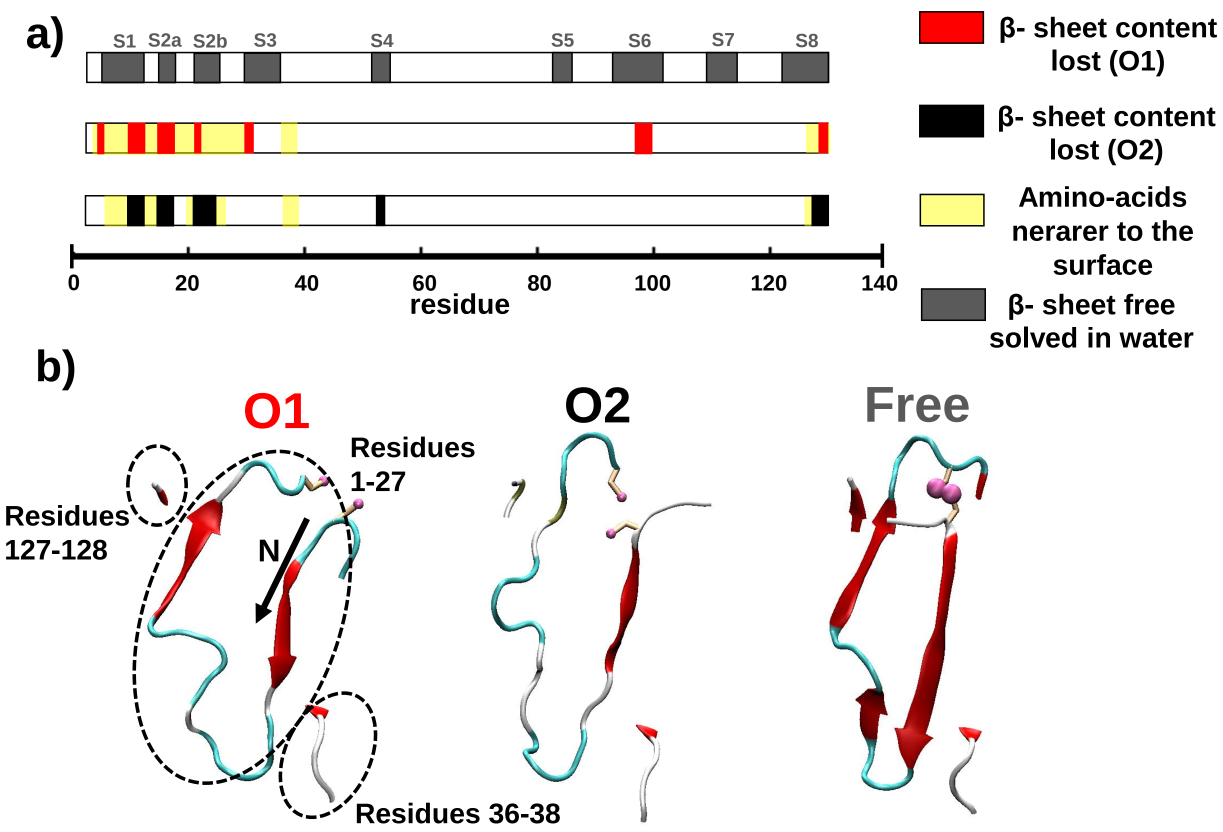


Figure S9: **Characterization of the wild-type regions where the $\beta - sheet$ content is lost during the adsorption simulation.** We focus on the two adsorption simulations where larger protein secondary structure losses are observed, i.e. using the O1 and O2 initial orientations (see Fig. 5 of the main manuscript). **a)** Amino-acids which have lost its $\beta - sheet$ structure at the end of the adsorption simulation for both the O1 (red) and O2 (black) cases. The amino-acids belonging to all the $\beta - sheets$ of the Azurin wild-type protein are also here shown for comparison (gray). **b)** Secondary structure content of the protein regions that are in contact with the surface (closer than 8Å) at the end of the adsorption process for the O1 (left panel) and O2 (center panel) initial orientations. These protein regions correspond to three different amino-acid segments: residues 1-27, residues 36-38 and residues 127-128 (marked with yellow color in **a)**). The secondary structure content of that amino-acid segments in the free solved state of the wild-type protein is also shown (right panel). From this figure, we conclude that most of the $\beta - sheet$ content lost during adsorption is located in the protein-surface contact regions. Additionally, we observe that the main difference between the O1 and O2 initial orientations is located in the S2b $\beta - sheet$ (residues 20-24), which is practically distorted in the latter case but not in the former.

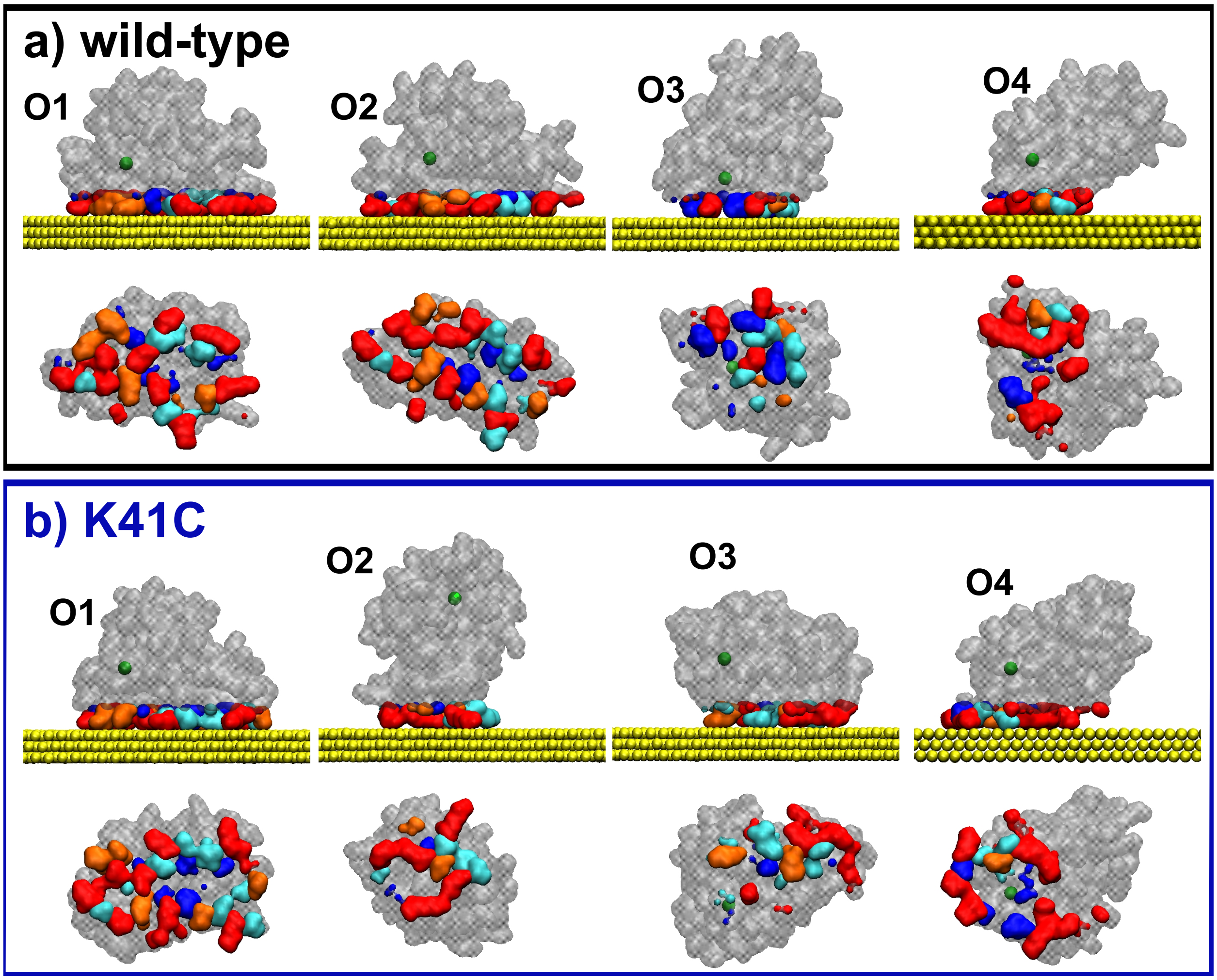


Figure S10: **Hydrophobic level description of the protein-surface contacting areas.** Here we show the side view (top panel) and bottom view (bottom panel) of the Azurin Connolly surface[1] for all the **a)**wild-type and **b)**K41C adsorption configurations. The bottom views are also represented in Fig. 5 (wild-type) and Fig. 6 (K41C) of the main manuscript. The protein-surface contacting residues are colored according to their hydrophobicity index: very hydrophobic (blue), hydrophobic (cyan), neutral (orange), and hydrophilic (red).The rest of the Azurin amino-acids (non-interacting) are colored in grey. The copper(II) ion is shown using its van-der-Waals representation in an opaque green color

# References

(1) Connolly, M. L. Analytical molecular surface calculation. *Journal of Applied Crystallography* **1983**, *16*, 548–558.
